# Revisiting the Formation of a Native Disulfide Bond: Consequences for Protein Regeneration and Beyond

**DOI:** 10.3390/molecules25225337

**Published:** 2020-11-16

**Authors:** Mahesh Narayan

**Affiliations:** The Department of Chemistry and Biochemistry, The University of Texas as El Paso, El Paso, TX 79968, USA; mnarayan@utep.edu; Tel.: +1-915-747-6614

**Keywords:** oxidative folding, regeneration, disulfide bond, native disulfide, native-like structure, protein misfolding, neurodegenerative disorders

## Abstract

Oxidative protein folding involves the formation of disulfide bonds and the regeneration of native structure (N) from the fully reduced and unfolded protein (R). Oxidative protein folding studies have provided a wealth of information on underlying physico-chemical reactions by which disulfide-bond-containing proteins acquire their catalytically active form. Initially, we review key events underlying oxidative protein folding using bovine pancreatic ribonuclease A (RNase A), bovine pancreatic trypsin inhibitor (BPTI) and hen-egg white lysozyme (HEWL) as model disulfide bond-containing folders and discuss consequential outcomes with regard to their folding trajectories. We re-examine the findings from the same studies to underscore the importance of forming native disulfide bonds and generating a “native-like” structure early on in the oxidative folding pathway. The impact of both these features on the regeneration landscape are highlighted by comparing ideal, albeit hypothetical, regeneration scenarios with those wherein a native-like structure is formed relatively “late” in the R→N trajectory. A special case where the desired characteristics of oxidative folding trajectories can, nevertheless, stall folding is also discussed. The importance of these data from oxidative protein folding studies is projected onto outcomes, including their impact on the regeneration rate, yield, misfolding, misfolded-flux trafficking from the endoplasmic reticulum (ER) to the cytoplasm, and the onset of neurodegenerative disorders.

## 1. Introduction

**Scheme 1:**
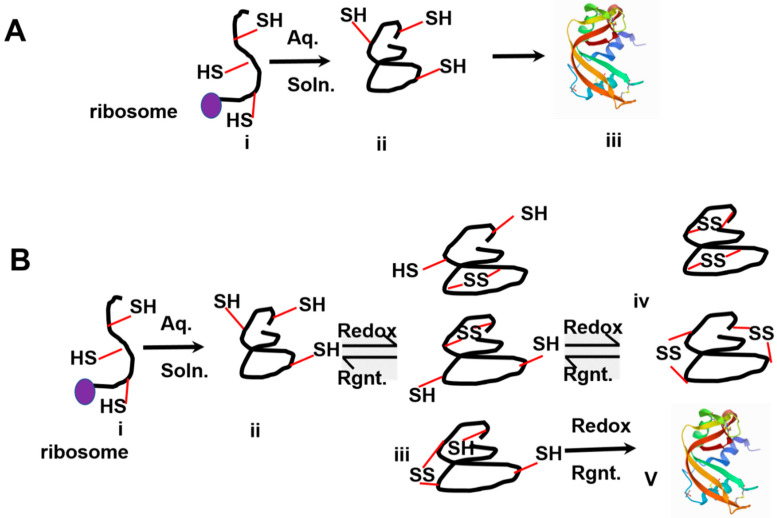
(**A**) Oxidative folding is a process by which the newly synthesized polypeptide undergoes both oxidation of its cysteines and the acquisition of disulfide bonds. (i) Protein synthesis from the ribosome (purple filled oval). (ii) Hydrophobic collapse in an aqueous medium. (iii) Native structure (N; PDBID: 7RSA). (**B**) Oxidative folding in vitro. (i) Disulfide bond-containing proteins synthesized from the ribosome (purple filled oval) are used to study oxidative folding pathways using in vitro techniques. The fully reduced protein (ii; R) acquires disulfide bonds in the presence of redox reagents to form 1S (iii) and 2S species (iv), which occur as an isomer ensemble. The native protein (N, PDBID: 7RSA; (v) is eventually generated.

Proteins that reside in the plasma membrane of cells, or are exported to extracellular domains, generally contain disulfide bonds. This unique covalent linkage lends stability to the protein in what might otherwise constitute rather harsh extracellular environments (such as the lumen of the stomach) [1,2,3]. Oxidative protein folding is used to describe the process of the regeneration of disulfide bond-containing proteins from their fully reduced and unfolded state to their native, biologically active form (Scheme 1A) [4,5,6,7,8,9,10,11,12,13,14,15,16,17,18,19,20,21,22,23,24,25]. The native structure is generally referred to as “N” and the fully reduced, unfolded protein is usually termed “R”. Usually, complete reduction of all disulfide bonds in a protein s in a loss of its structure. The term “U”, which refers to the unfolded state in protein folding and unfolding studies, is a special case in oxidative protein folding. It represents a species that is unfolded, but retains its native disulfide bonds. However, a key difference between U and R is that the former state requires harsh conditions such as low pH, chemical denaturants, or extreme temperature. On the other hand, while R initially requires a denaturant and a reducing agent to unfold the protein and reduce its disulfide bonds, respectively, the anaerobic removal of these reagents does not result in the reacquisition of structure (as would be the case with U) [4,5,6]. Furthermore, structurally, unlike U, R is collapsed. It can best be described as a statistical coil. This is because hydrophobic interactions along with other intramolecular enthalpic interactions prevent it from adopting a so-called “random coil” conformation [26,27,28,29,30].

During regeneration, the fully reduced polypeptide is observed to undergo gradual oxidation via thiol-disulfide exchange reactions with the help of external redox reagents such as glutathione (GSSG/GSH), dithiothrreitol (DTT^ox^/DTT^red^), molecular oxygen or other synthetic catalysts, including those derived from selenium [29,30,31,32,33,34,35,36,37,38,39,40]. During this process, a number of intermediate species that vary in the number of their disulfide bonds are observed in the oxidative folding landscape [4,5,6,41]. Since the process involves the oxidation of cysteines to form disulfide-bonds, there is a reduction in the number of free cysteines and a concomitant increase in disulfide bonds in the protein with the progress of time (Scheme 1B). Depending on the number of disulfide bonds in the intermediates, they are termed 1S (one disulfide-bond containing), 2S (two disulfide-bond containing), 3S, ……. *n*S (*n* disulfide bond-containing) species/intermediates. Most intermediates in oxidative folding, and particularly those that appear early on in the process, are unfolded (unstructured) [4].

The formation of structure is integral to oxidative folding [5,6,14,17,24]. As will be discussed using model protein folding studies, structure alone permits the locking of native disulfide bonds and the “removal” of intermediates from an equilibrium ensemble of unstructured disulfide bond-containing folding intermediates. At this juncture, it suffices to state that the formation of structure in the regenerative process may occur anywhere along the oxidative folding trajectory [4,5,6]. However, wherever the structure-forming step might occur, it is extremely impactful and therefore, the earlier that it occurs in the regeneration trajectory, the more productive it is for the regeneration rate and yield.

## 2. General Considerations

The oxidative folding pathways of several proteins have been studied in detail [41,42,43,44,45,46,47,48,49,50]. We recapitulate the oxidative folding trajectories of a few “model” proteins and discuss key features pertaining to their respective regenerative pathways. It is to be noted that post ribosomal synthesis, regeneration of disulfide bond-containing proteins occurs in the endoplasmic reticulum (ER) [51,52,53,54]. Here, the “oxidizing” environment is enabled by a favorable ratio of GSSG/GSH and promotes disulfide bond formation. Furthermore, in the ER, the oxidoreductase chaperone protein disulfide isomerase (PDI) plays a pivotal role in the processing of disulfide bonds and cysteines. The role of PDI in oxidative folding has been extensively discussed elsewhere [55,56,57,58,59].

**Scheme 2:**
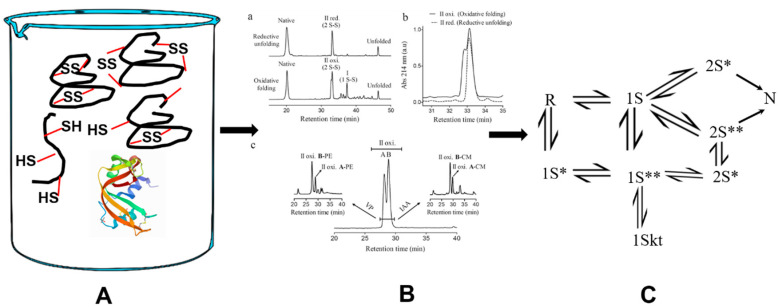
The oxidative folding mixture (**A**) is periodically withdrawn and sampled using a variety of analytical tools (**B**; HPLC). The intermediates, separated by HPLC, are quantified as a function of time. (**B**) **a**: The reversed-phase HPLC chromatograms of Nerita VersiColor carboxypeptidase inhibitor two-disulfide intermediates. **b**: Comparison of oxidative folding and reductive unfolding by superimposition using an intermediate of the protein. **c**: The fraction corresponding to the intermediate is further deconvoluted to at least by two species. Further details can be found in the corresponding reference (https://doi.org/10.1038/s41598-017-05657-7). By analyzing the formation and consumption (to form other products) of all intermediates as a function of time, a scheme can be derived for the pathway by which the protein regenerates (**C**). Furthermore, 1S**, 2S* and 2S** are one and two-disulfide-bond-containing intermediates, respectively, with native-like structure. 1Skt is a one-disulfide-bond-containing kinetically-trapped species. The HPLC image (**B**) has been adapted from https://doi.org/10.1038/s41598-017-05657-7 in accordance with http://creativecommons.org/licenses/by/4.0/.

To initiate oxidative folding, the protein of interest is first introduced into a reducing and unfolding buffer (6 M GdnHcl, 5 mM DTT^red^, 100 mM Tris-HCl, 1 mM EDTA pH 8) for a period of ~10–30 min [26,35,36,41,44,52]. The reducing and denaturing agents are separated from the now fully reduced and unfolded protein (R) using column chromatography or dialysis (against a low pH buffer which prevents acquisition of disulfide bonds). R can be stored indefinitely if lyophilized; or for a few days at 4 °C in a low pH solution (such as dilute acetic acid). Oxidative folding is usually initiated by placing R into a folding buffer (pH 7–8, 100 mM Tris-HCl, 1 mM EDTA) under anaerobic conditions. Aliquots are periodically withdrawn and the regeneration process stopped by a variety of techniques such as (i) reducing the pH; (ii) alkylating the thiols; (iii) freezing the mixture. The aliquots are then analyzed by a variety of techniques including HPLC, enzymatic assays, CD spectroscopy or by NMR to detect, resolve and quantify the intermediates and detect N. By measuring the formation of both intermediates and N as a function of time, a detailed map of the folding pathway and the folding kinetics can be constructed (Scheme 2).

## 3. The Oxidative Folding Pathways of Disulfide-Bond-Containing Proteins

### 3.1. Bovine Pancreatic Ribonuclease A (RNase A)

RNase A is a four-disulfide-bond-containing protein with native disulfide bonds at positions (26–84), (40–95), (58–110) and (65–72). Its primary role is the cleavage of RNA into smaller fragments. This process permits the cell to eliminate unwanted RNA [60,61,62,63,64].

**Figure 1:**
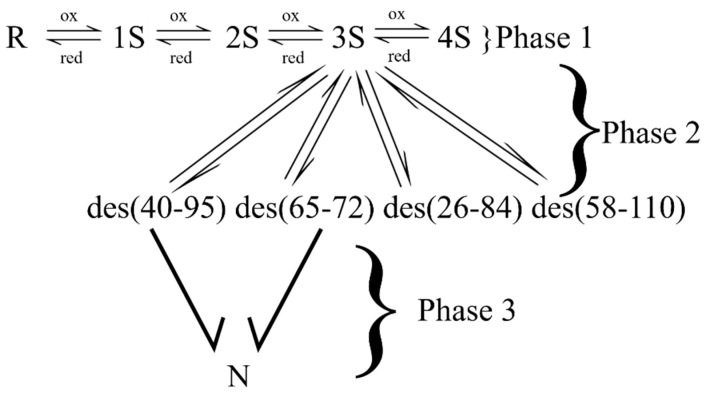
The oxidative folding pathway of RNase A (at 5 °C). In Phase I, unstructured intermediates form a quasi-equilibrium distribution. The structure is formed from 3S intermediates in a conformational folding step (Phase 2). The native protein (N) is formed upon the oxidation of des (40–95) and des (65–72) in Phase 3. The two other des species remain kinetically trapped.

The regeneration of RNase A has been extensively studied using DTT^ox/red^ [65,66,67,68,69,70]. The choice of this redox reagent mitigates the formation of mixed disulfides between protein thiols and the redox reagent which would otherwise lead to an increase in the complexity of the folding landscape relative to GSH/GSSG. The fully reduced polypeptide containing eight free cysteines (26, 40, 58, 65, 72, 84, 95, 110) is sequentially oxidized to form 1S, 2S, 2S and 4S species (Figure 1) [65].

In the absence of mixed disulfides between the redox reagent and the protein thiols, there are 28 possible 1S isomers in an eight-cysteine protein, i.e., the 1S ensemble contains a theoretical maximum of 28 one-disulfide-bond-containing species that differ in disulfide connectivity. In practice, not all isomers may populate the regeneration space because the distribution of the species (disulfide-connectivities) within this ensemble is driven by both entropic and enthalpic interactions [71,72]. That is, cysteines that reside farther away from each other in the primary sequence have a greater entropic cost associated with their oxidation to disulfide bonds. For example, formation of the (26–110) disulfide bond would be entropically unfavorable relative to the formation of (65–72). Its fractional concentration, within the 1S ensemble, would therefore be expected to be less than that of the (65–72) bond. Nevertheless, favorable enthalpic interactions among amino acids near the cysteines or elsewhere in the polypeptide chain may overcome the entropic cost of forming disulfides and increase the fractional concentration of entropically unfavorable linkages [73,74,75].

The species within the 1S ensemble are in equilibrium with each other [4,5,6,65,66,67,68,69,70]. In the absence of structure, disulfide-bonds within each 1S isomer freely reshuffle via thiol-disulfide exchange reactions with any of the six available cysteines. In turn, the 1S ensemble is in *quasi-equilibrium with the 2S ensemble (210 isomers), which is in quasi-equilibrium with the 3S ensemble (420 isomers). The equilibrium within an ensemble is independent of the redox reagent whereas the quasi-equilibrium between ensembles is redox reagent dependent.

*Note: The term quasi-equilibrium denotes the fact that the R-4S species is slowly consumed via the formation of “N”; an irreversible process [4,5,6]. Thus, the equilibrium between R-4S continuously, albeit slowly, shifts away from R, and therefore is termed “quasi-equilibrium”.

The formation of the steady-state or quasi-equilibrium distribution is relatively rapid in RNase A (assuming reasonable redox conditions) relative to the formation of N. None of the species within the steady-state distribution is structured and, as a result, thiol–disulfide exchange reactions freely take place within *intermediates and, between the intermediates and the redox couple.

*Note: It is assumed that the solution is sufficiently diluted to avoid thiol–disulfide exchange reactions between nS and (n + 1)S intermediates.

In RNase A, the formation of structure occurs in the 3S ensemble. Here, two unstructured 3S species containing the native linkages (26–84, 40–95, 58–110) and (26–84, 65–72, 58–110) conformationally fold to form two 3S* species (Figure 1) [69,70]. The * is used to denote “structure”. The two 3S* intermediates in RNase A are des (65–72) and des (40–95). The formation of structure is a key feature that discriminates intermediates from their unstructured counterparts. Importantly, it results in the “removal” of the two intermediates from the quasi-equilibrium/steady-state distribution of species (R→4S), i.e., the equilibrium between 3S and each of the des species lies to the product side. This is because, structure protects the formed (native) disulfide bonds from undergoing any thiol-disulfide exchange reactions such as intramolecular reshuffling or reduction. Furthermore, it orients the remaining cysteines for easy oxidation and facilitates the formation of N.

Des (65–72) and des (40–95) are “native-like” in structure. They have some enzymatic activity and thermal transitions of ~40 °C by contrast to N, which exhibits a thermal transition at 65 °C [69,70].

The formation of N takes place by the oxidation of cysteines (40–95) and (65–72) in des (40–95) and des (65–72), respectively. The formation of N is deemed irreversible.

When regeneration is performed at low temperatures (15 °C), two other structured species are observed in the oxidative folding milieu (Figure 1) [76,77]. Des (26–84) and des (58–110) with melting points of ~18 °C are observed to form from their unstructured 3S counterparts. Unlike des (65–72) and des (40–95), des (26–84) and des (58–110) are kinetically trapped. Their buried cysteines are inaccessible to the redox reagent and they linger in the regeneration landscape for “days”. The two kinetically trapped intermediates slowly disappear from the regeneration landscape by reconverting to their 3S intermediates, to 3S* or N [76]. The direct conversion to N is only possible via local unfolding events that can expose buried cysteines for oxidation while preserving the formed native disulfides.

### 3.2. Bovine Pancreatic Trypsin Inhibitor (BPTI)

**Figure 2:**
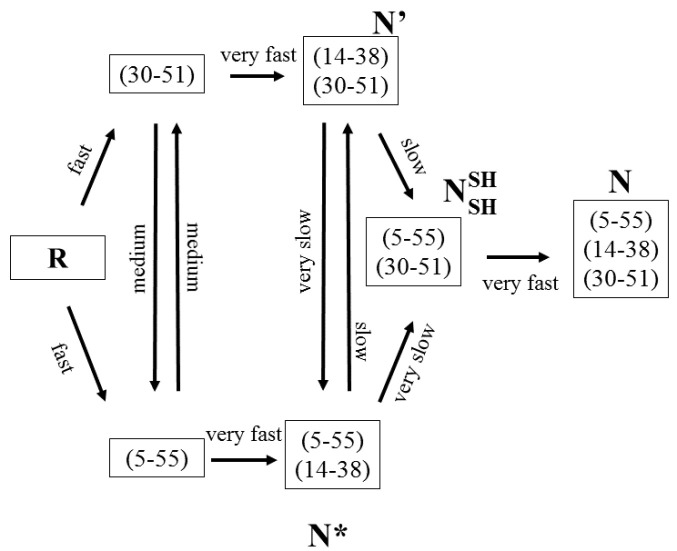
The oxidative folding pathway of BPTI. The fully reduced protein rapidly forms two one-disulfide-bond-containing species, viz. (30–51) and (5–55), both of which are native bonds. Both species are very rapidly oxidized to form two structured, albeit kinetically trapped, 2S* intermediates. Each species undergoes intramolecular disulfide rearrangement to form des (14–38) which is rapidly oxidized to form the native protein. N* and N’ are two-disulfide-bond-containing, kinetically-trapped, intermediates with native-like structure. N is the native protein which contains three native disulfide bonds with the connectivities as shown.

BPTI contains three disulfide bonds at positions (5–55), (14–38) and (30–51). The fully reduced protein is consumed to form two transient 1S intermediates by the oxidation of cysteines 30 and 51 and 5 and 55 (Figure 2) [78,79,80,81,82,83,84]. The second step involves the rapid oxidation of surface-accessible cysteines 14 and 38 to form des (5–55) and des (30–51). Both these species are somewhat kinetically trapped, in that the remaining disulfide bond in each des species is buried. Therefore, they only slowly interconvert to des (14–38) via intramolecular thiol–disulfide exchange reactions. The oxidation of the 2S, des (14–38) to N ((5–55), (30–51), (14–38)) takes place rapidly via oxidation of the surface-exposed cysteines, 14 and 38.

### 3.3. Hen-Egg White Lysozyme

This four-disulfide-bond-containing protein, with native linkages at positions (76–94), (64–80), (6–127) and (30–115), is highly aggregation-prone. As a result, it also serves as a model for unraveling amyloid-forming trajectories. The oxidative folding pathway of lysozyme can be studied under highly dilute conditions, as has been done by several groups [85,86,87,88,89,90,91,92].

**Figure 3:**
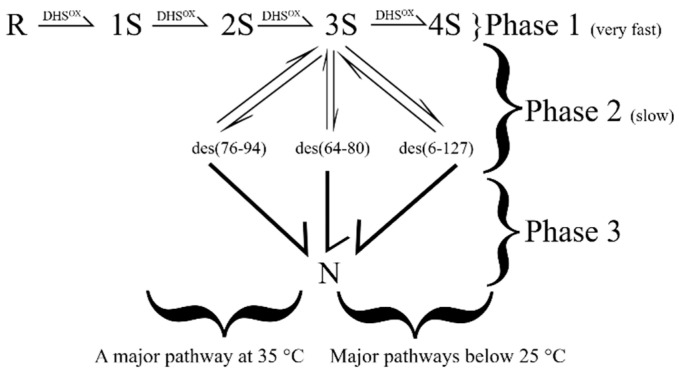
The oxidative folding pathway of HEWL. In Phase I, unstructured intermediates are rapidly formed. Structure is acquired in phase 2 via the concomitant oxidation and conformational folding of native-disulfide-bond-containing 2S species to form three 3S* intermediates. N HEWL is formed from the oxidation of all three 3S* species, albeit with differences in flux.

In the presence of 1.0 mM GSH/0.2 mM GSSG, the fully reduced protein equilibrates to form 1S and 2S intermediates within a few minutes of initiating regeneration (Figure 3). The formation of structure arises via the oxidation of native-disulfide-bond-containing 2S intermediates to form three 3S* species, viz. des (76–94), des (64–80) and des (6–127). The formation of N takes place by the oxidation of the remaining pair of cysteines in each des species.

## 4. The Role of Structure in Oxidative Folding

A survey of the regeneration landscape of model proteins, including RNase A, BPTI and HEWL, reveals the presence of both unstructured intermediates and the eventual appearance of native-like structure (via structured intermediates) that leads to the formation of N [4,5,6,41,44]. From these findings and the structural studies of native-like intermediates, it has become known that the formation of native/native-like structure requires that *two criteria be met: (i) the exclusive presence of native disulfide bonds is necessary and (ii) a critical number of native disulfide bonds are required. These conditions are necessary, but not sufficient. For example, in RNase A, the formation of 3S* from 3S also requires the X-Pro peptide bonds at the four prolines to be in the correct isomeric state [93,94,95,96,97,98]. However, towards the end of this review, we will discuss alternative lines of evidence where structure precedes, and drives, native disulfide bond formation.

*Note: Of course, if R itself is capable of forming a native/native-like structure under folding conditions, it follows then that disulfide bonds are (somewhat) redundant [99,100,101,102].

The propensity to form R to form a native-like structure was recently examined in the three-disulfide-bond-containing μ-concotoxins [103]. In this elegant study, the disulfides were selectively mutated out to infer their contribution to the structure and the stability of the toxin [103]. The work revealed that the individual disulfides make differing contributions to the stability of the native fold and the range of their contributions is such that they lie between the extreme models of BPTI and Hirudin vis-à-vis their contribution to structure acquisition.

Once the necessary criteria for acquisition of native/native-like structure have been met, the acquisition of native-structure is a key event that drastically impacts the regeneration rate and yield [4,5,6]. For example, in RNase A, structure does not appear until the 3S stage. In the interim, R has been partially consumed to form 1S through 4S species and the quasi-equilibrium distribution of species persists until a useful outlet to N is generated via the formation of the two native-like intermediates. Up until that point, all species in the regeneration landscape simply undergo thiol-disulfide exchange reactions which include oxidation, reduction and reshuffling reactions and the regeneration rate is essentially “0”. These “back-and-forth” reactions are essentially futile past the point of forming, perchance, native disulfide bonds. As previously discussed, this is because non-native disulfide bonds do not permit the formation of native/native-like structure; only native disulfides can. Thus, any delay in forming structure from those unstructured intermediates that possess native disulfide linkages simply prolongs the regeneration process and reduces the regeneration rate. It also makes the protein susceptible to aggregation. These aspects are discussed below in detail.

## 5. The Impact of Futile vs. Fruitful Reactions on the Regeneration Trajectory

**Scheme 3:**
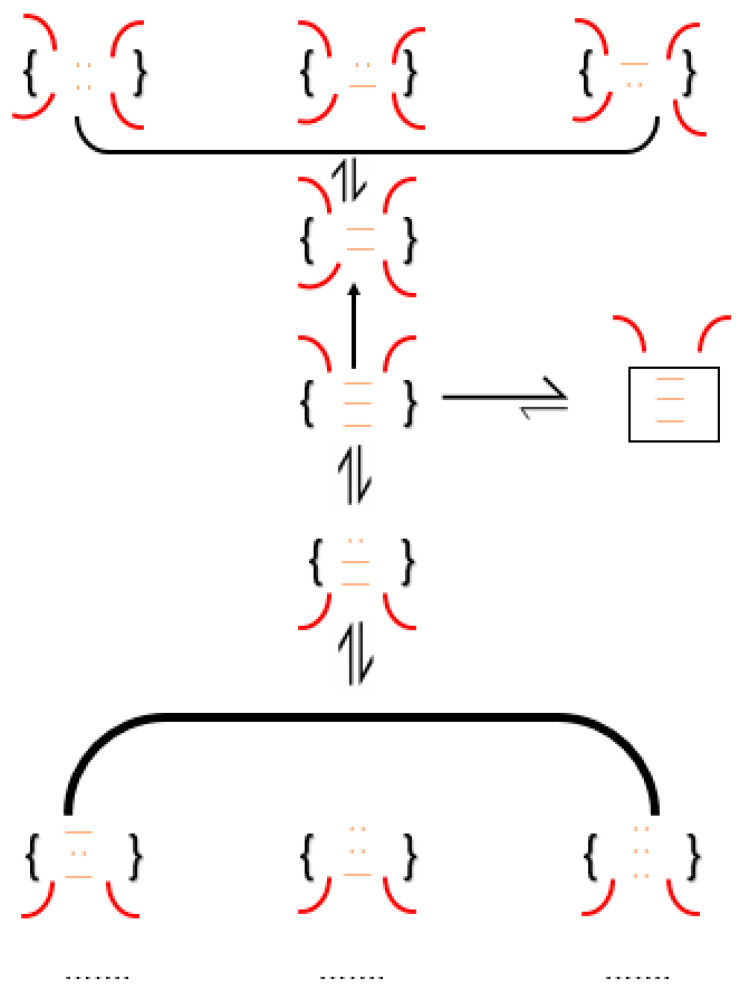
The fate of a three-disulfide-bond-containing (3S) unstructured species that is poised to fold. Towards the right is the 3S to 3S* structure-forming transition which is fruitful. Competing with this are reshuffling reactions which overwhelmingly produce statistically favored non-native disulfide bonds and a reduction reaction to produce a native disulfide-bond-containing 2S species. The 2S species itself can then rapidly reshuffle to form an overwhelming majority of non-native disulfide-bond-containing isomers. Red dots (. .), non-native disulfide bonds; straight red line (^___^), native disulfides; curved red line (( ), thiolates.

Consider an unstructured species in RNase A with the disulfide linkages at positions (26–84), (58–110) and (40–95). At a pH of 8, and at room temperature, thiol–disulfide exchange reactions are relatively rapid [65]. Therefore, the aforementioned species has the following possible fates (Scheme 3) [52,53]: (i) conformationally fold to form 3S*; (ii) reshuffle to form an unstructured 3S isomer; (iii) become reduced to form a 2S species. Scenarios (ii) and (iii) are futile. They not only succeed in delaying the regeneration of N, but also increase the chance of aggregation. Furthermore, both reactions can outcompete the conformational folding process in RNase A (which is proline isomerization-dependent and, therefore, slow). Of the competing processes, the reshuffling of the native-disulfide-only 3S species to its non-native disulfide-bond-containing isomer is the most rapid (at pH 8 and room temperature). The process is not dependent on external redox reagents. Furthermore, the “effective concentration” of the intramolecular thiolate far exceeds any possible concentration of redox reagent that can be added. Finally, the formed non-native disulfide can continue to rapidly reshuffle to form one of many possible 3S isomers that now contain two non-native disulfides, and so on.

Simply put, the statistical (random) probability of forming non-native disulfide bonds far exceeds that of forming native disulfide bonds. For example, in RNase A, of the 420 3S species possible, only four contain native disulfide bonds. Thus, the statistical occurrence of a 3S species containing only native disulfides is 4/420 or <1%. Finally, the newly formed 3S species can become oxidized to 4S, placing itself even further away from acquiring native-like structure.

A similar argument can be made for the product in scenario (iii) where the 2S intermediate can rapidly reshuffle to form isomers with the non-native linkages—yet again reinforcing the futility of interactions (chemical and or/physical) that do not promote native/native-like structure.

Therefore, the “securing” of native disulfide-bonds, beyond the statistically probable population, is only possible by the acquisition of structure [4,5,6]; a key event that protects and preserves the native disulfide bond from “degenerating” into futile species. Conversely, native/native-like structure can arise only from species exclusively possessing native disulfide bonds. Therefore, early enthalpic interactions that overcome any entropic costs associated with forming native disulfide bonds are invaluable in increasing the probability that a conformational folding event can occur and preserve those native disulfides [52,53,99,100], subsequently removing those species from the equilibrium of thiol–disulfide exchange reactions and promoting regeneration and native yield.

## 6. The Formation of Native Disulfide Bonds and Structure in Other Disulfide-Bond-Containing Proteins

The oxidative folding pathway of BPTI results in the very rapid formation of two 1S species, viz. (5–55) and (30–51) [78,79,80,81,82,83,84]. Both species possess some structure. Furthermore, intermediates retaining the (30–51) bond populate one branch of bifurcated its regeneration pathway. The second trunk also possesses exclusively native linkages. Importantly, the formation of the native bond early on facilitates the formation of structure which, in turn, seeds that species in the regeneration pathway. It must be noted that fully reduced BPTI is compact [81]; a feature that may assist in skewing the proportion of native disulfide bonds early on. The resulting outcome is the rapid formation of two 2S* species, viz. des (5–55) and des (30–51), while simultaneously limiting the formation of non-native disulfide-bond-containing intermediates [80,83]. In BPTI, however, as previously discussed, conversion of both 2S* species to N is not possible as the remaining pair of cysteines remains buried in the native structure. We will revisit this aspect later on. Both des species undergo a local unfolding reaction, which serves to partially preserve one native disulfide bond in each des species, followed by reshuffling to form des (14–38), the third structured 2S* species. This newly formed des (14–38) is rapidly oxidized to N.

The regeneration pathway features of HEWL lie between that of BPTI and RNase A [85,86,87,88,89,90,91,92]. The oxidation of 2S to 3S* species is critical in preventing 3S and 4S species from populating the regeneration landscape. Prior to that, one observes non-native disulfide-containing species in the form of unstructured 1S and 2S ensembles. Unlike the RNase A pathway which populates 1S-4S intermediates, the HEWL landscape is relatively limited in the futile intermediates to 1S and 2S species. This, in turn, favors the efficiency of the regeneration and facilitates a higher yield.

## 7. The Impact of Native Disulfide-Bonds and Early Structure Acquisition in Oxidative Protein Folding

The information learnt from these aforementioned “model” oxidative folders permits us to make some general observations: (1) the formation of native disulfide bonds is key to the successful formation of a native/native-like structure; (2) the formation of a native/native-like structure early on in the trajectory limits fruitless thiol–disulfide exchange reactions and the formation of unproductive non-native disulfide bond-containing intermediates. We can test the impact of these observations on the oxidative folding trajectory through relatively simple, back-of-the-envelope-type calculations.

**Scheme 4:**
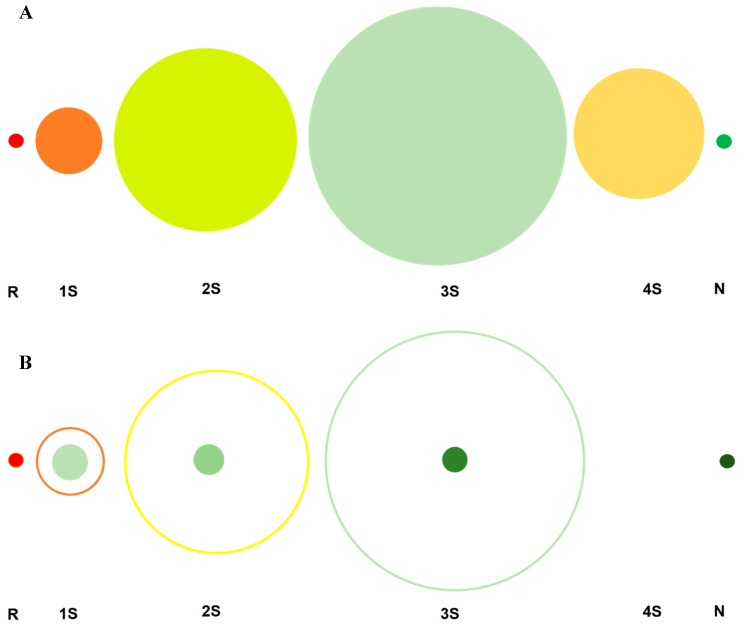

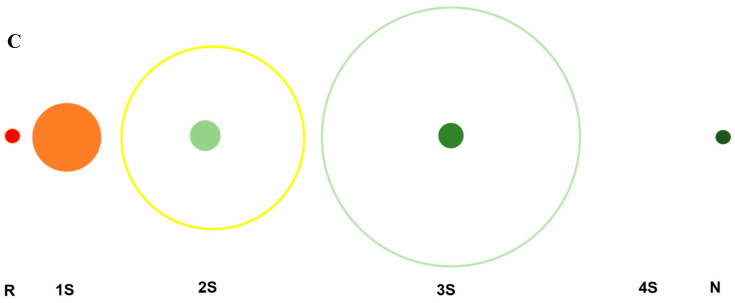
(**A**). The experimentally observed distribution of intermediates in the oxidative folding pathway of a four-disulfide-bond-containing proteins, such as RNase A, using a redox reagent that does not form mixed disulfides. The number of chemically distinct species in each ensemble are represented by the area under the circles. R = 1; 1S = 28; 2S = 210; 3S = 420; N = 1. (**B**). An idealized oxidative folding trajectory where only native disulfide bond-containing intermediates are populated in a four-disulfide-bond-containing protein. Here, R = 1; 1S = 8; 2S = 6; 3S = 4; N = 1. The difference in area between each filled circle and the open circle reveals the “efficiency” of this hypothetical pathway. Since the four 3S species directly oxidize to N, the 4S ensemble is absent. (**C**). An oxidative folding trajectory where only native disulfide bond-containing intermediates are populated in the 2S ensemble of a four-disulfide-bond-containing protein. The 28 species in the 1S ensemble funnel into the 6 possible native disulfide bond-containing species of the 2S ensemble. The 6 species then form 4 3S species (only native bonds) before forming N. The “efficiency” of this hypothetical pathway lies between those in Scheme 4A,B, since there is the formation of futile, non-native disulfide bond-containing species that must reshuffle to structure- enabling, native disulfide-bond-containing intermediates. Lastly, akin to the pathway in 4B, since the four native disulfide bond-containing 3S species directly oxidize to N, the 4S ensemble is absent.

In Scheme 4A shows the experimentally observed distribution of intermediates that populate the oxidative folding pathway of a four-disulfide-bond-containing protein such as RNase A. R, a chemically single species rapidly gets consumed to form several intermediates differing in disulfide-bond number and content viz., 28 1S species, 210 2S species, 420 3S species and 104 4S species prior to forming native-like 3S* species and N. Scheme 4B, by contrast, is an idealized (or highly desired) pathway that traverses a trajectory exclusively populating native disulfide bonds. The ideal pathway is superimposed over the experimentally observed pathway. In the hypothetical pathway, the initially formed native disulfide bonds are preserved, and “passed onward”, by “structural factors”; a scenario that is possible if a structure is acquired early on in the oxidative folding trajectory, including R. Such a structure, however loose, would be crucial in aligning native-bond-forming cysteines such that a native disulfide bond would be formed with high probability. An examination of the statistical distributions of species in the “ideal pathway” clearly demonstrates the advantage for a protein to exclusively populate native disulfide bonds and acquire a native/native-like structure early on. For example, only eight 1S species would be present in a system sampling only native disulfide bonds versus 28 in the experimentally observed pathway; and so on. The attenuation in the number of species, coupled with the fact that these species are “productive”, would accelerate oxidative maturation. Furthermore, and importantly, the generation of a native-like structure, early in the regeneration pathway, would play an even greater role. For example, if a native-like structure emerges in R and persists in the 1S ensemble, it would help facilitate correct alignment of the remaining cysteines such that rapid oxidation to form native disulfide would dominate any other possible competing reaction, such as intramolecular thiol-disulfide-exchange or reduction, further preserving the native-disulfide-only pattern. Ideally, a once native-like structure is acquired, oxidative regeneration would simply become a “stapling exercise”; an exercise in “click” chemistry. Essentially, in this mechanism, R is diluted, or distributed, into a very narrow set of highly productive intermediates; a process that not only conserves the folding momentum but also accelerates it.

We contrast the “ideal” scenario with a “less ideal”, but nevertheless “still highly desirable” scenario (In Scheme 4C). The same four-disulfide-bond-containing protein lets us assume that native-like structure is formed by oxidation and simultaneous conformational folding of the native-disulfide-bond-containing 1S species to form the 2S*. This would restrict the 2S* species to six species and the 3S ensemble to four 3S* species, as it would curtail the onward trajectory by exclusively populating native disulfide-containing species (due to alignment of the remainder of the cysteines, as previously discussed).

The outcome would be a relatively collimated trajectory (post the 1S ensemble) via the reduction in the 2S ensemble from 210 to 6 species and that in the 3S ensemble from 420 species to four 3S* species. It would also limit the formation of 4S species since the 3S* species would be able to directly oxidize to N.

In both hypothetical schemes (Scheme 4B,C), the net result would be a reduction in the number of unstructured species, restrictions in fruitless thiol–disulfide exchange reactions and a higher efficiency in the rate of formation of N.

## 8. Shortcomings: The Kinetically Trapped Species

**Scheme 5:**
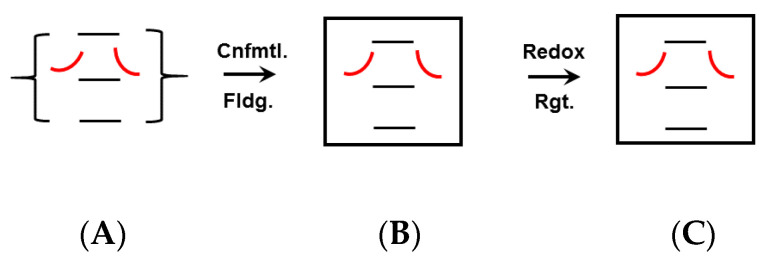
The conformational folding of a formation of an unstructured native disulfide bond-containing intermediate (**A**) to form a structured-intermediate with buried cysteines (**B**). The latter tends to be kinetically trapped as structure occludes the buried cysteines from becoming disulfides. The presence of redox reagents has no impact on the species (**C**).

While it appears that the predominance of native disulfide bonds and the formation of a native-like structure early in the regeneration pathway is extremely favorable, several practical observations reveal pitfalls that limit the regeneration rate and yield, even when native disulfide bonds and native-like structure are formed in the intermediates. For example, in both RNase A and BPTI, the formation of particular native disulfide bonds prior to the formation of other native disulfide bonds leads to the formation of kinetically trapped species [76,104]. As previously discussed, in RNase A, des (58–110) and des (26–84) are kinetically trapped and in BPTI, the formation of the (14–38) bond leads to des (5–55) and des (30–51), both 2S* species, that are kinetically trapped. Inspection of the crystal structures of both proteins reveals the cause for an intermediate with native disulfide bonds becoming kinetically trapped. (26–84) and (58–110) in RNase A, and (5–55) and (30–51) in BPTI are buried disulfide disulfide bonds. While the formation of buried native disulfides is always desired, a delay in their formation prior to the formation of the relatively externally located native disulfide bonds results in the acquisition of a native structure and the burial of cysteines within the structure. In both proteins, the pairs of cysteines responsible for the formation of (26–84) and (58–110) disulfide bond in RNase A, and the (5–55) and (30–51) disulfide bond in BPTI, lie buried within the protein. Thus, the formation of surface-exposed disulfide bonds prior to the oxidation of buried cysteines results in the “closing-off” of the protein to redox reagents (Scheme 5).

The buried cysteines, though aligned for oxidation, remain reduced and the species becomes kinetically trapped.

Kinetically trapped intermediates compromise regeneration rates. They need to undergo either local or global unfolding to expose the buried cysteines to the redox couple. The unfolding event makes them susceptible to both intramolecular thiol–disulfide exchange and to reduction reactions in addition to aggregation. Therefore, in an ideal regeneration scenario, native disulfide bonds need to form “inside-out”. Such a mechanism would limit the formation of kinetically trapped species while restricting non-native disulfides while forming a structure. Even in such an “inside-out” mechanism, the structure cannot be so rigid as to occlude redox reagents from oxidizing the pre-aligned cysteines.

## 9. Oxidative Folding in Biology

Protein folding and oxidative regeneration is an important biological process with consequences that can not only lead to loss of function but also trigger pathology [105,106]. Similar to the scenario in conformational folding, there always exists a competition between folding and aggregation during the regeneration of disulfide bond-containing proteins [4,106]. Delayed acquisition of the native structure, be it due to proline-isomerization dependency or the statistically low probability of populating native disulfide bonds (a precursor to conformational folding), makes the biopolymer susceptible to aggregation via continued exposure of its hydrophobic residues. An atomic-level understanding of physico-chemical forces driving oxidative protein folding is a cornerstone for advancing our understanding of their roles in biology, the onset of myriad misfolding-related pathologies and successful intervention therein [107,108,109,110,111,112].

Disulfide bond-containing proteins constitute approximately 15% of the human proteome [113,114]. Among secreted proteins, that number reaches 65%. Interestingly, more than 50% of proteins involved in protein misfolding diseases, or amyloidosis, contain disulfide bonds including the prion protein, superoxide dismutase 1 (involved in ALS), tau (taupathies and Alzheimer’s disease), islet amyloid polypeptide (IAPP; diabetes) and HEWL (systemic amyloidosis). Native disulfide bonds regulate fibril-forming trajectories by slowing down the time to mature fibril formation. They regulate fibrillar morphology. A molecular examination reveals that the mechanism of inhibition of fibril-formation lies in the position of disulfide bonds such that they prevent the aggregation-prone conformation of some proteins (insulin, IAPP, β-lactoglobulin, and lysozyme) [113,114,115]. In some cases, the covalent bonds directed specificity to non-covalent intermolecular interactions via domain-swapping [114].

The thiol-disulfide oxidoreductase PDI has not only been found to be upregulated in ALS models of mice but was also found to colocalize with inclusion bodies in the motor neurons of the G93A mice model of SOD1 and in human ALS patients [113,114]. Conversely, PDI overexpression protects against mutant SOD1 neurotoxicity while knocking down of PDI increased mutant SOD1 aggregation. PDI levels have also been found to be upregulated in PD, AD, and prion-diseased brains. These co-relations further suggest that disulfide chemistry may be implicated in the pathogenesis of the aforementioned PMDs. Corruption of PDI itself can lead to an increase in the flux of misfolded proteins emerging from the ER [52,53]. The exodus from the ER can lead to the overburdening of the parkin-ubiqutin machinery, delaying the ubiquitination of cytosolic misfolding-prone proteins such as α-synuclein and creating a “traffic jam” of misfolded debris awaiting proteosomal recycling [116,117]. Outcomes include corruption of neuronal/cellular homeostasis, apoptosis, and the onset to neurodegenerative pathology.

## 10. Learnings from Oxidative Protein Folding

Importantly, oxidative folding studies have advanced our understanding of protein folding “reactions”. By the availing of the ability to tune the thiol–disulfide exchange rate (via changes in pH, mutagenesis, choice of redox reagents and temperature), regeneration studies of proteins have permitted researchers to sufficiently slow down the protein folding reaction to permit analysis of atomic-level interactions that drive the process. Oxidative protein folding has been successfully exploited to isolate native-like intermediates along the folding trajectory. Comparison of the structural studies of the intermediates with the native protein provides information about the interatomic interactions that guide the folding process. The study of kinetically trapped intermediates in oxidative folding has parallels with kinetically trapped intermediates in conformational folding. The kinetic trapping secludes the protein into a soluble conformation and thereby inhibits aggregation [118]. The trapped intermediate can then convert to the native fold via a local unfolding event that keeps secure all other native (-like) intermolecular interactions (or native disulfide disulfide bonds). Albeit slow, it paves a pathway that circumvents global unfolding and the risk of aggregation (fruitless thiol-disulfide exchange).

Finally, there has been much discussion on whether disulfide bonds precede structure formation or whether structure guides the acquisition of native disulfide bonds [12,119,120]. In experimental studies of systems with sufficient disulfide complexity such as RNase A and HEWL, there has been no evidence of structure beyond compaction in intermediates with one or more non-native disulfide bonds. In simpler systems, say with one disulfide and no free cysteines, one might imagine that the processes of structure-acquisition and disulfide-bond-formation may be concomitant; or that structure may precede cysteine oxidation. In vitro, if structure precedes disulfides, there are examples proving that cysteines get occluded from redox reagents and the intermediate becomes kinetically trapped. In vivo, the availability of the oxidoreductase PDI to anchor a mixed disulfide intermediate, and simultaneously serve as the redox couple, provides a pathway by which structure can precede folding.

All said, there may be more “malleability” in the folding trajectory that previously imagined [121]. The intracellular pathways, aided by chaperones such as PDI and peptidyl prolyl isomerases, may differ from reductionist-derived processes. Nevertheless, the compendium of studies performed to-date in “test-tubes” and the techniques advances therein have provided a wealth of data on protein folding from which core principles underlying the U/R→N transformation have been derived [122,123]. Finally, other aspects of disulfide bonds have come to the forefront with regard to their impact on protein function and disease onset. Disulfide bonds being chiral, a possibility that handedness in the disulfide bond of prions may transmit stereochemical information that can influence the manner of folding or refolding into pathogenic forms has been discussed [124]. The impact of conformational isomerization of disulfide bonds in protein dynamics and, therefore, function has been probed by NMR line-shape analysis of its cysteine carbon peaks and by techniques such as residual dipolar coupling (RDC) [125,126]. In other research addressing the role of disulfides in influencing the conformational dynamics of proteins, it was found that the introduction of single disulfides spanning a large portion of the lysozyme shifted both the structure and dynamics of hydrophobic core residues of the protein [127]. In a recent landmark study that utilized a combination of biophysical and computational techniques (mass spectrometry, theoretical simulations, dynamic nuclear polarization-enhanced solid-state nuclear magnetic resonance and cryo-electron microscopy), it was demonstrated that thiol groups of cysteines undergo chemical mutation to form S-glutathionylated and S-nitrosylated adducts and impacted the formation non-native disulfide bonds [128]. This covalent chemical modification was found to occur prior to chain release, supported by arguments that since the ribosome exit tunnel provides sufficient space even for disulfide bond formation, it is not surprising to detect these chemo-mutated variants. Finally, it is pointed out that students and researchers interested in a predictive examination of the formation of disulfide bonds can utilize “DISULFIND” [129]. This is a user-friendly platform that assigns the native disulfide bonds in a protein based on sequence alone.

In sum and substance, it is beneficial for structure to precede disulfide bond formation, as seen in the advantageous offered through hypothetical trajectories in Scheme 4.; and, it remains the most viable option as long as cysteines can become oxidized to cement the bond. The ultimate goal, whatever the process, is to avoid aggregation and pathogenic outcomes.
